# Supplementary material for: Strategic adjustment of parental care in tree swallows: life-history trade-offs and the role of glucocorticoids
Source: R Soc Open Sci. 2016 Dec 14;3(12):160740. doi: 10.1098/rsos.160740 (PMC5210693; doi:10.1098/rsos.160740)

Supplementary Materials for Akcay, Lendvai et al. Strategic adjustment of parental care in tree swallows: life-history trade-offs and the role of glucocorticoids. *in review* for Royal Society Open Science

Table S1. Model selection table for the effect of time period, population (pop), and treatment on female feeding rates. The offset for duration of observations was included in all models.

| Model | period | pop | treatment | period*pop | period*treatment | df | AICc | ΔAICc | weight |
| --- | --- | --- | --- | --- | --- | --- | --- | --- | --- |
| 1 | + | + |  | + |  | 10 | 2196.5 | 0 | 0.373 |
| 2 | + | + | + | + | + | 14 | 2196.8 | 0.29 | 0.322 |
| 3 | + | + |  |  |  | 7 | 2198.2 | 1.7 | 0.16 |
| 4 | + |  |  |  |  | 6 | 2198.4 | 1.9 | 0.144 |

Table S2. Model selection table for the effect of stage and treatment on cort levels. Plus signs indicate that the predictor variable was included in the model.

| Model | pop | stage | treatment | stage*treatment | df | AICc | ΔAICc | weight |
| --- | --- | --- | --- | --- | --- | --- | --- | --- |
| 1 |  | + | + | + | 7 | 616.1 | 0 | 0.339 |
| 2 |  | + | + |  | 6 | 616.4 | 0.28 | 0.295 |
| 3 | + | + | + | + | 8 | 617.2 | 1.11 | 0.194 |
| 4 | + | + | + |  | 7 | 617.4 | 1.34 | 0.173 |

Table S3: The model selection table and the model averaged estimate for the effect of treatment and population on clutch size. The only variable included in the averaged model was treatment.

|  | treatment | df | AICc | ΔAICc | weight |
| --- | --- | --- | --- | --- | --- |
| Model 1 |  | 3 | 518.9 | 0 | 0.703 |
| Model 2 | + | 4 | 520.6 | 1.72 | 0.297 |
| Model averaged estimate (SE) | 0.046 (0.07) |  |  |  |  |
| *p* | 0.53 |  |  |  |  |

Table S4: The model selection table and the model averaged estimate for the effect of treatment and population on brood size on day 6. The only variable included in the averaged model was treatment.

|  | treatment | df | AICc | ΔAICc | weight |
| --- | --- | --- | --- | --- | --- |
| Model 1 |  | 4 | 519.1 | 0 | 0.682 |
| Model 2 | + | 5 | 520.7 | 1.53 | 0.318 |
| Model averaged estimate (SE) | 0.061 (0.08) |  |  |  |  |
| *p* | 0.43 |  |  |  |  |

Table S5: The full model on clutch size (GLMM with Poisson distribution and log link) with fixed factors treatment, population and their interaction, and relative lay date (number of days from the first egg of the respective population) as a random factor. N=76 nests

|  | Estimate | St Error | p value |
| --- | --- | --- | --- |
| treatment | 0.028 | 0.14 | 0.84 |
| population | 0.0048 | 0.14 | 0.97 |
| treatment*population | 0.016 | 0.20 | 0.93 |

Table S6: The full linear mixed model on average nestling size on day 6, with fixed factors treatment, population and their interaction, and relative lay date (number of days from the first egg of the respective population) as a random factor. N=70 nests

|  | Estimate | | St Error | | p-value |
| --- | --- | --- | --- | --- | --- |
| treatment | -0.29 | 0.68 | | 0.67 | |
| population | 2.14 | 0.69 | | 0.0030 | |
| treatment*population | 0.96 | 0.99 | | 0.33 | |

Table S7: The full linear mixed model on average nestling size on day 12, with fixed factors treatment, population and their interaction, and relative lay date (number of days from the first egg of the respective population) as a random factor. N=64 nests

|  | Estimate | St Error | p value |
| --- | --- | --- | --- |
| treatment | -1.23 | 1.05 | 0.25 |
| population | 2.89 | 1.09 | 0.01 |
| treatment*population | 2.73 | 1.58 | 0.09 |

Table S8: The full model on number of fledged chicks (GLMM with Poisson distribution and log link) with fixed factors treatment, population and their interaction, and relative lay date (number of days from the first egg of the respective population) as a random factor. N=75 nests

|  | Estimate | St Error | p value |
| --- | --- | --- | --- |
| treatment | 0.19 | 0.18 | 0.31 |
| population | -0.02 | 0.19 | 0.90 |
| treatment*population | -0.11 | 0.28 | 0.69 |

Figure S1: Clutch size depending on population and treatment. The boxplots are as in the main text.


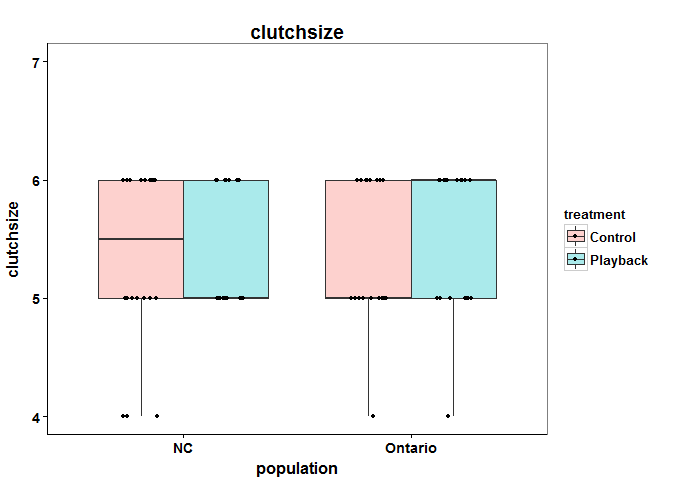

Figure S2: Nestling mass on day 6 depending on population and treatment. Nestlings on day 6 were heavier in Ontario than nestlings in North Carolina.


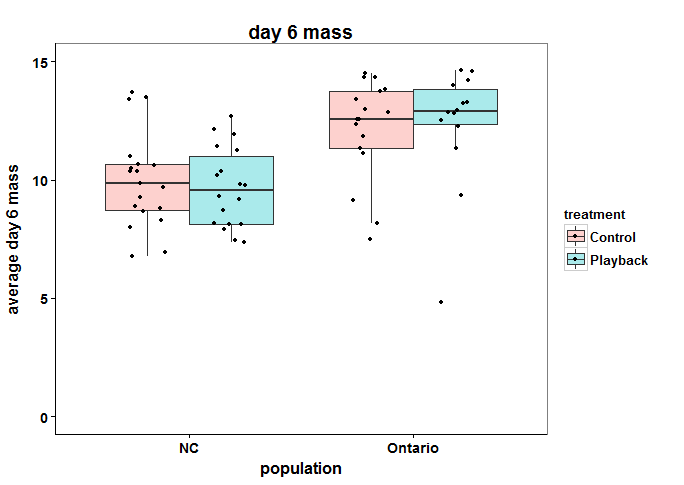


Figure S3: Nestling mass on day 12 depending on population and treatment. Nestlings on day 12 were heavier in Ontario than nestlings in North Carolina.


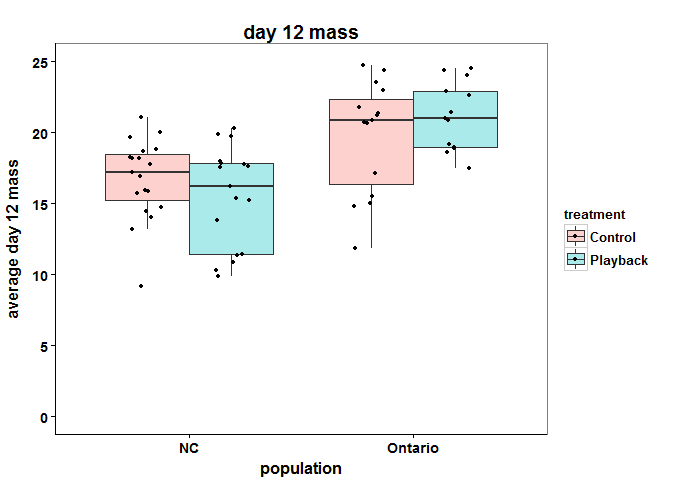


Figure S4: Number of nestlings fledged depending on population and treatment.
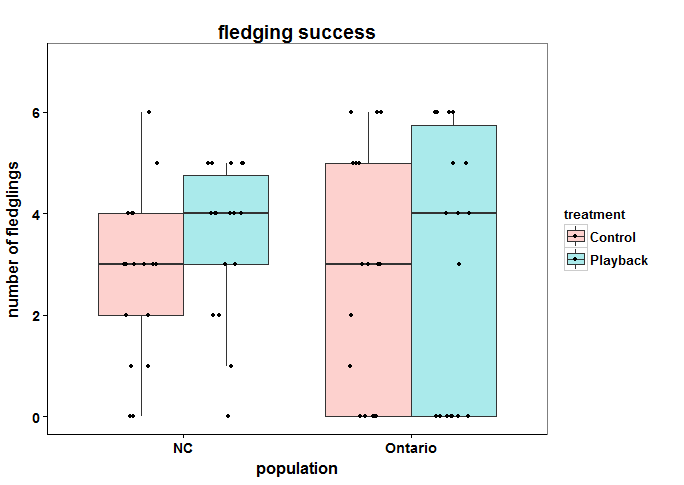

Supplement: Supplementary tables and analyses [file rsos160740supp2.docx]
